# Polyphenolic compounds appear to limit the nutritional benefit of biofortified higher iron black bean (*Phaseolus vulgaris* L.)

**DOI:** 10.1186/1475-2891-13-28

**Published:** 2014-03-26

**Authors:** Elad Tako, Steve E Beebe, Spenser Reed, Jonathan J Hart, Raymond P Glahn

**Affiliations:** 1 USDA/ARS, Robert W. Holley Center for Agriculture and Health, Cornell University, Ithaca, NY 14853, USA; 2 CIAT- International Center for Tropical Agriculture, Cali, Colombia; 3 Department of Plant Biology, Cornell University, Ithaca, NY 14853, USA

**Keywords:** Beans, Biofortification, Iron bioavailability, *In vitro* digestion/Caco- 2 cell model, Broiler chicken, Intestine

## Abstract

**Background:**

Our objective was to determine if a biofortified variety of black bean can provide more bioavailable-iron (Fe) than a standard variety. Two lines of black beans (*Phaseolus-vulgaris* L.), a standard (DOR500; 59μg Fe/g) and biofortified (MIB465; 88μg Fe/g) were used. The DOR500 is a common commercial variety, and the MIB465 is a line developed for higher-Fe content. Given the high prevalence of Fe-deficiency anemia worldwide, it is important to determine if Fe-biofortified black beans can provide more absorbable-Fe.

**Methods:**

Black bean based diets were formulated to meet the nutrient requirements for the broiler (*Gallus-gallus*) except for Fe (dietary Fe-concentrations were 39.4±0.2 and 52.9±0.9 mg/kg diet, standard vs. biofortified, respectively). Birds (n=14) were fed the diets for 6-weeks. Hemoglobin-(Hb), liver-ferritin and Fe-related transporter/enzyme gene-expression were measured. Hemoglobin-maintenance-efficiency and total-body-Hb-Fe values were used to estimate Fe-bioavailability.

**Results:**

Hemoglobin-maintenance-efficiency values were higher (P<0.05) in the group consuming the standard-Fe beans on days 14, 21 and 28; indicating a compensatory response to lower dietary-Fe. Final total-Hb-Fe body content was higher in the biofortified vs. the standard group (26.6±0.9 and 24.4±0.8 mg, respectively; P<0.05). There were no differences in liver-ferritin or in expression of DMT-1, Dcyt-B, and ferroportin. *In-vitro* Fe-bioavailability assessment indicated very low Fe-bioavailability from both diets and between the two bean varieties (P>0.05). Such extremely-low *in-vitro* Fe-bioavailability measurement is indicative of the presence of high levels of polyphenolic-compounds that may inhibit Fe-absorption. High levels of these compounds would be expected in the black bean seed-coats.

**Conclusions:**

The parameters of Fe-status measured in this study indicate that only a minor increase in absorbable-Fe was achieved with the higher-Fe beans. The results also raise the possibility that breeding for increased Fe-concentration elevated the levels of polyphenolic-compounds that can reduce bean Fe-bioavailability, although the higher levels of polyphenolics in the higher-Fe beans may simply be coincidental or an environmental effect. Regardless, Fe-biofortified beans remain a promising vehicle for increasing intakes of bioavailable-Fe in human populations that consume high levels of these beans as a dietary staple, and the bean polyphenol profile must be further evaluated and modified if possible in order to improve the nutritional quality of higher-Fe beans.

## Introduction

Iron (Fe) deficiency is the most common nutrient deficiency worldwide [1]. Iron deficiency is particularly widespread in low-income populations where consumption of meat is low and consumption of cereal grains and legumes which contain inhibitors of iron absorption make up the bulk of a diet with little diversity [2-7]. Thus, a major cause of Fe deficiency is low bioavailability from plant-based diets containing Fe bioavailability inhibitors such as polyphenols [4-9]. In the past, policies aimed to alleviate Fe deficiency anemia have primarily involved the use of dietary Fe supplementation for at-risk populations, food fortification, and diversification of diets. However, these strategies can be difficult to sustain in resource-poor regions due to cost, limited access to health care, the partial availability of centralized food processing facilities required for post-harvest crop fortification, and other limiting logistical factors [10-12].

Progress has been made during the last decade to develop cereal and legume lines with a higher Fe content and/or bioavailability [5-9,13-15]. This strategy is known as biofortification [16]. In theory, this effort is expected to improve the Fe status of humans in developing countries as it utilizes the tools of plant breeding and agriculture to enhance the amount of absorbable Fe in staple food crops. Once these lines have been developed and stabilized for traits such as higher Fe concentration and or higher Fe bioavailability, then the nutritional benefits should be driven and provided by agriculture and thus sustainable in terms of cost and benefit.

It was previously shown that the common bean (*Phaseolus vulgaris* L), provides significant quantities of protein and energy and is a source of vitamins and minerals including Fe [7,8,17,18]. The common bean is an attractive candidate for Fe biofortification because there is genetic variability of Fe concentration and therefore it is possible to breed for significant increases in Fe concentrations in beans [6-8,19]. Also, Fe concentrations in beans are high relative to the cereals and have the potential to deliver substantially more Fe. In studies with rats, bean genotypes with high Fe concentrations provided more absorbable Fe than genotypes with lower concentrations of Fe [7,8,15,19]. However, rats are known to be very efficient at absorbing Fe relative to humans, hence these studies have questionable relevance to human nutrition [5-9,19].

In recent years, scientists at CIAT (Centro Internacional de Agricultural Tropical, Cali, Colombia) have developed Fe-biofortified black beans that consistently contain 85–100 μg Fe/g bean (dry basis), a substantial increase over normal beans which tend to range between 50–60 μg Fe/g bean [5,6,8,20]. In a previous study using piglets as test subjects, this same high Fe black bean variety provided more Fe for hemoglobin synthesis in Fe deficient pigs than a standard black bean line [5]. This result demonstrated that Fe biofortified beans can enhance Fe status in Fe- deficient pigs even when fed as part of a complete diet where the difference in Fe concentration between the diets was only 12 μg/g and the feeding period was only 5 weeks [5]. In addition, previous studies using poultry have demonstrated that increasing Fe concentrations in large-seeded, red-mottled beans by about 25 μg/g provides more bioavailable and therefore absorbable Fe *in vitro* and *in vivo*[8]. Also it was concluded that increased Fe concentration can limit the polyphenolic inhibitory effect on Fe absorption provided that polyphenolic content remains constant as Fe content increases [8]. Indeed the combination of a poultry model and an *in vitro* system such as the in vitro digestion Caco-2 cell model have been demonstrated to be effective and efficient analytical tools for testing common bean varieties showing significant differences in bioavailability [5-9,15,21].

The objective of the current study was to compare the capacities of biofortified and standard, black bean lines to deliver Fe for Hb synthesis using a Fe deficient broiler chickens as the *in vivo* model. If these screening tools indicate that nutritional benefits exist, then human efficacy studies using these lines would be warranted and can proceed with greater confidence of success.

## Materials and methods

### Animals, diets and study design

Sixty Cornish cross fertile broiler eggs were obtained from a commercial hatchery (Moyer’s chicks, Quakertown, PA). The eggs were incubated under optimal conditions at the Cornell University Animal Science poultry farm incubator. Upon hatching (hatchability rate was 92%), chicks were allocated into 2 treatment groups on the basis of body weight, gender and blood hemoglobin concentration (aimed to ensure equal distribution between groups, n = 14): 1. “Standard Fe”: 40% black bean diet (39 μg Fe/g); 2. “Biofortified Fe”: 40% black bean diet (53 μg Fe/g). Experimental diets had no supplemental Fe. Diets compositions are shown in Table 1.

**Table 1:** Composition of experimental diets

**Ingredient**	**Standard bean diet**	**Biofortified bean diet**
	**g/kg (by formulation)**	
Biofortified Beans (88 μg Fe/g)	_	400
Standard Beans (59 μg Fe/g)	400	_
Corn	350	350
Corn oil	30	30
Dry skim milk	100	100
Corn starch	46.75	46.75
Vitamin/mineral premix (no Fe) ^1^	70	70
_DL_-Methionine	2.5	2.5
Choline Chloride	0.75	0.75
Total (g)	1000	1000
Selected components	mean ± SEM, n = 5 (by analysis)	
Fe (μg Fe/g) ^2^	39.4 ± 0.2^b^	52.9 ± 0.9^a^
Phytate:Fe molar ratio^3^	8.25 ± 0. 65^a^	8.95 ± 0.72^a^

^1^Vitamin and mineral premix provided/kg diet (330002 Chick vitamin mixture; 235001 Salt mix for chick diet; *Dyets* Inc. Bethlehem, PA).

^2^Iron concentrations in the diets were determined by an inductively-coupled argon-plasma/atomic emission spectrophotometer (ICAP 61E Thermal Jarrell Ash Trace Analyzer, Jarrell Ash Co. Franklin, MA) following wet ashing.

^3^Method for determining phytate contents are described in the materials and methods section.

^a,b^Within a row, means without a common letter are different, P < 0.05.

Chicks were housed in a total-confinement building (1 chick per 0.5 m^2^ metal cage). Birds were under indoor controlled temperatures and were provided 16 h of light. Each cage was equipped with an automatic nipple drinker and manual self-feeder. All birds were given *ad libitum* access to water (Fe content was 0.379 ± 0.012 μg Fe/ml). Feed intakes were measured daily (as from day 1). Iron intakes were calculated from feed intakes and Fe concentration in the diets.

The two black bean lines used in the study were a commercial variety for South America (DOR500, Low-Fe) and a biofortified line (MIB465, High-Fe). Seed was multiplied in Palmira, Colombia under fertile soil conditions and standard agronomic practices and shipped to Ithaca, New York in sealed containers imported as grain. Upon arrival, beans were rinsed in ultra-pure (18Ω) water and then cooked (autoclaved for 45 minutes in water and until soft). Beans were then freeze-dried and milled prior to the mixing the diets (for all processing stainless steel appliances were used).

### Blood analysis and hemoglobin (Hb) measurements

Blood samples were collected weekly from the wing vein (n = 14, ~100 μL) using micro-hematocrit heparinized capillary tubes (Fisher, Pittsburgh, PA). Samples were collected in the morning following an 8 h overnight fast. The samples were analyzed for hemoglobin (Hb) concentration (see below). Body weights and hemoglobin concentrations were measured weekly.

Fe bioavailability was calculated as hemoglobin maintenance efficiency (HME) [5-9]:

$$\;\mathrm{HME}=\frac{\mathrm{Hb}\;\mathrm{Fe},\;\mathrm{mg}\;\left(\mathrm{final}\right)-\mathrm{Hb}\;\mathrm{Fe},\;\mathrm{mg}\;\left(\mathrm{initial}\right)}{\mathrm{Total}\;\mathrm{Fe}\;\mathrm{Intake},\;\mathrm{mg}}\times \;100$$

Where Hb-Fe (index of Fe absorption) = total body hemoglobin Fe. Hb-Fe was calculated from hemoglobin concentrations and estimates of blood volume based on body weight (a blood volume of 85 mL per kg body weight is assumed) [5-9,15,22]:

$$\begin{array}{l} \text{Hb}-\text{Fe}\left(\text{mg}\right)=B.W.\left(\text{kg}\right)\times 0.085\;L\;\text{blood}/\mathrm{kg}\\ \;\times \;\text{Hb}\left(g/L\right)\times 3.35\text{mg}\;\text{Fe}/g\;\text{Hb}. \end{array}$$

Fe intakes were calculated from feed intake data and Fe concentrations in the feed.

At the end of the experiment (day 42nd), birds were euthanized by carbon dioxide exposure. The digestive tracts and livers were quickly removed from the carcass and separated into various sections for tissue (small intestine and liver ~ 1-2 cm; ~2-3 g, respectively). The samples were immediately frozen in liquid nitrogen, and then stored in a -80°C freezer until analysis.

All animal protocols were approved by the Cornell University Institutional Animal Care and Use Committee.

Blood Hb concentrations were determined spectrophotometrically using the cyanmethemoglobin method (H7506-STD, Pointe Scientific Inc. Canton, MI) following the kit manufacturer’s instructions.

### Isolation of total RNA

Total RNA was extracted from 30 mg of duodenal (proximal duodenum) tissue using Qiagen RNeasy Mini Kit (Qiagen Inc.,Valencia, CA) according to the manufacturer’s protocol. All steps were carried out under RNase free conditions. RNA was quantified by absorbency at 260–280 nm. Integrity of the 28S and 18S rRNA was verified by 1.5% agarose gel electrophoresis followed by ethidium bromide staining.

### DMT1, DcytB and ferroportin gene expression analysis

As previously described [5-9,15,20,22], PCR was carried out with primers chosen from the fragments of chicken (*Gallus gallus*) duodenal and hepatic tissues [DMT1 gene (GeneBank database; GI 206597489) (forward: 5′-AGC CGT TCA CCA CTT ATT TCG-3′; reverse: 5′-GGT CCA AAT AGG CGA TGC TC-3′), DcytB gene (GI 20380692) (forward: 5′-GGC CGT GTT TGA GAA CCA CAA TGT T-3′; reverse: 5′-CGT TTG CAA TCA CGT TTC CAA AGA T-3′) and Ferroportin gene (GI 61098365) (forward: 5′-GAT GCA TTC TGA ACA ACC AAG GA′; reverse: 5′-GGA GAC TGG GTG GAC AAG AAC TC-3′)]. Tissue-specific 18S rRNA was used to normalize the results [(GI 7262899) (forward: 5′- CGA TGC TCT TAA CTG AGT-3′; reverse: 5′-CAG CTT TGC AAC CAT ACT C-3′)]. All PCR products were separated by electrophoresis on 2% agarose gel, stained with ethidium bromide, and quantified using the Quantity One 1-D analysis software (Bio-Rad, Hercules, CA).

### *In-vitro* iron bioaccessibility assessment

An *in vitro* digestion/Caco-2 cell culture model [5-9,21,23,24] was used to assess *in vitro* Fe bioavailability. With this method, the cooked bean samples and the formulated diets were subjected to simulated gastric and intestinal digestion. Exactly 0.5 g of the freeze dried cooked beans and diets samples were utilized for each replication of the in vitro digestion process.

### Harvesting of Caco-2 cells for ferritin analysis

The protocols used in the ferritin and total protein contents analyses of Caco-2 cells were similar to those previously described [5-9,22,24]. Caco-2 cells synthesize ferritin in response to increases in intracellular iron concentration. Therefore, we used the ratio of ferritin/total protein (expressed as ng ferritin/mg protein) as an index of the cellular Fe uptake. All glassware used in the sample preparation and analyses was acid washed.

### Ferritin and Fe in the liver, electrophoresis, staining and measurement of gels

Liver ferritin and liver Fe quantification were conducted as previously described [7-9,22,25,26]. The gels were scanned with Bio-Rad densitometer. Measurements of the bands were conducted using the Quantity-One 1-D analysis program (Bio-Rad, Hercules, CA). All tests were conducted in duplicates for each animal (n = 6).

### Polyphenol concentrations in diets

The list of reported compounds was generated by comparing calculated masses of flavonoids and hydroxycinnamic acids reported to be present in common bean [27] with high accuracy mass/charge (m/z) data derived from analysis of black bean seed coats using our Ultra Performance Liquid Chromatography/ Mass Spectrometry (UPLC/MS) system and related software. The identities of matching compounds were confirmed by comparison with UPLC retention times of standards and were sorted by relative abundance using Waters Metabolynx® software. Eight of the nine most abundant polyphenols indicated by this method were selected for quantitative comparison by the use of UPLC/MS and standard curves. Identification and quantification of the ninth (and second most abundant, possibly myricetin 3-o-glucoside), was not possible because there was no commercially available standard.

#### Polyphenol extraction

To one gram of ground bean material, 5 mL of methanol:water (50:50) was added. The slurry was vortexed for one minute, placed in a sonication water bath for 10 minutes, vortexed again for one minute, and centrifuged at 4000 x g for 15 minutes. The supernatant was filtered with a 0.45 μm syringe filter and stored for later use in a -20°C freezer.

#### LC/MS analysis

Extracts were analyzed by LC-MS with an Acquity UPLC coupled to a Xevo G2 QTof spectrometer (Waters Corp. Milford, MA). For LC analysis, 5 μL samples of extract were injected and passed through a HSS C18 1.8 μm 2.1 × 100 mm column (Waters®) at 0.4 mL/min. The mobile phase consisted of water with 0.1% formic acid (solvent A) and acetonitrile with 0.1% formic acid (solvent B). Polyphenols were eluted using linear gradients of 97.6 to 80% A in 2.5 min, 80 to 60% A in 0.5 min, 60 to 48% A in 2 min, 48 to 5% A in 0.5 min, 5 to 97.6% A in 1 min, and a 0.5 min hold at 97.6% A. Electro Spray Ionization (ESI) mass spectrometry was performed in positive ionization mode with a scan speed of 5 scans/sec in the mass range from 50 to 1200 Da. Lock-mass correction was used, with leucine-enkephalin as the external lock-mass standard. LC-MS data were analyzed by MassLynx® software.

### Phytic acid analysis in diets

Dietary phytic acid (phytate)/total phosphorus was measured as phosphorus released by phytase and alkaline phosphatase, following the kit manufacturer’s instructions (n = 5) (K-PHYT 12/12, Magazyme International, Ireland).

### Statistical analysis

Results were analyzed by ANOVA using the general linear models procedure of SAS software® (SAS Institute Inc. Cary, NC). Differences between treatments were compared by Tukey’s test and values were considered statistically different at P < 0.050 (values in the text are means ± SEM).

## Results

### Growth rates, hemoglobin (Hb), Hb Fe and Hb maintenance efficiency (HME)

**Figure 1:**
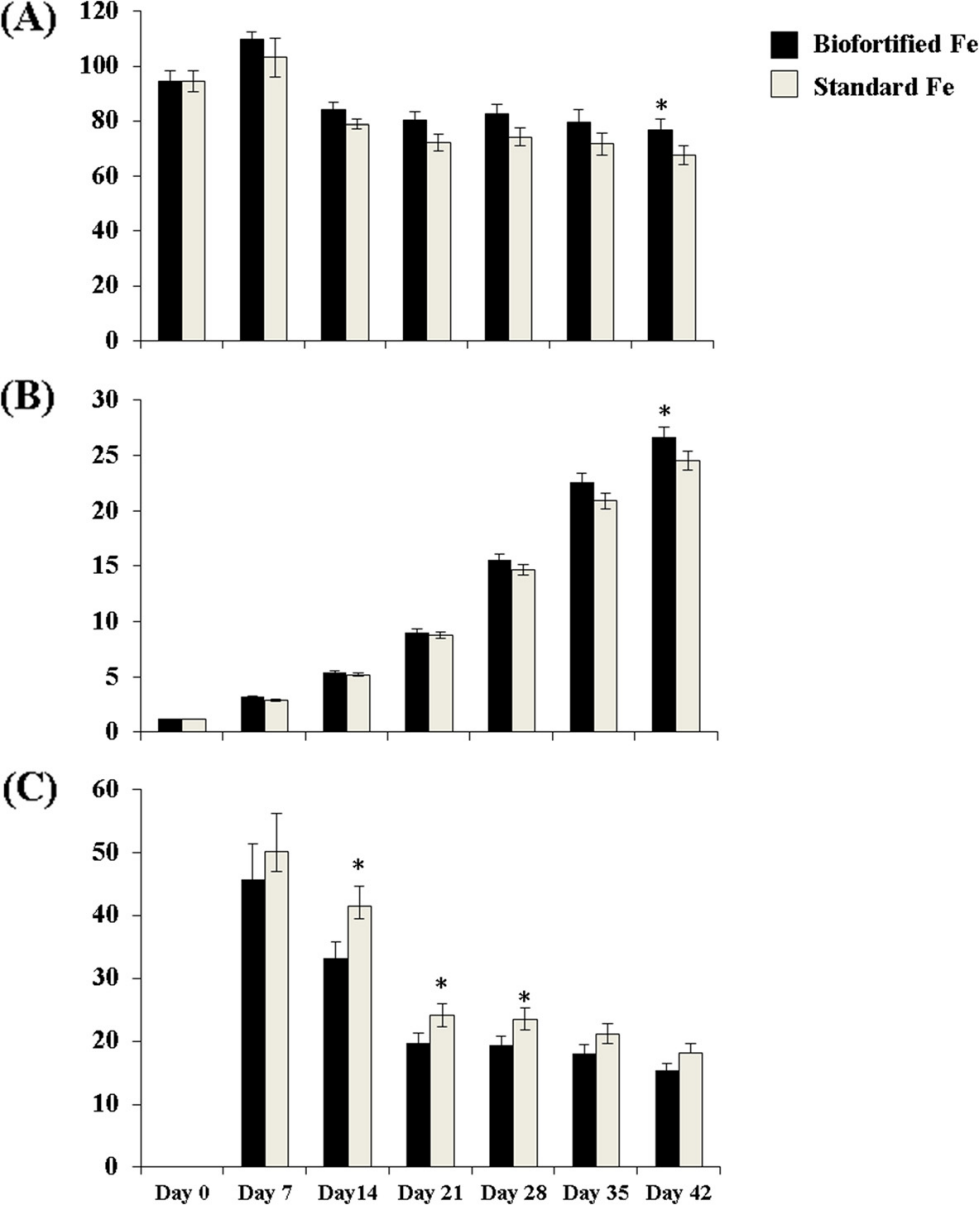
**(A) Hemoglobin (g/L), (B) Total body Hb Fe content (mg), and (C) Hemoglobin maintenance efficiency (HME, %), in chicken fed the tested diets from d 0 to d 42**^**1**^.^*^P < 0.05. ^1^Values are means ± SEM, n = 14. ^2^Values are mean daily feed intakes for the 7 days proceeding the day designated in the column heading. ^3^Values are mean ± SEM (cumulative weekly from day 0). ^4,5^Fe bioavailability was calculated as hemoglobin maintenance efficiency (*HME*) [5-9,15]: $\mathrm{HME}=\frac{\mathrm{Hb}\;\mathrm{Fe},\;\mathrm{mg}\;\left(\mathrm{final}\right)-\mathrm{Hb}\;\mathrm{Fe},\;\mathrm{mg}\;\left(\mathrm{initial}\right)}{\mathrm{Total}\;\mathrm{Fe}\;\mathrm{Intake},\;\mathrm{mg}}\times 100.$ Where Hb-Fe = total body hemoglobin Fe. Hb Fe was calculated from hemoglobin concentrations and estimates of blood volume based on body weight (a blood volume of 85 mL per kg body weight is assumed) [5-9,15,22]: Hb - Fe(mg) = B. W. (kg) × 0.085 L blood/kg  ×  Hb (g/L) × 3.35 mg Fe/g Hb.

There were no significant differences in feed intakes at any time throughout the study; thus, Fe intakes were consistently higher in the “Biofortified Fe” group vs. “Standard Fe” group (cumulative Fe intakes at week 6 were 166.8 ± 17.8 mg and 128.2 ± 15.6 mg, for the “Biofortified Fe” group vs. “Standard Fe” group, respectively). Hemoglobin concentrations tended to be higher in the “Biofortified Fe” group vs. “Standard Fe” group, and was significantly higher at week 6 (P < 0.05; Figure 1). Significant differences were measured in HME on days 14, 21 and 28 of the experiment between the “Biofortified Fe” group vs. “Standard Fe” group (Figure 1). In addition, the increase in total body Hb Fe values at the end of the 6th week was significantly greater in the “Biofortified Fe” group vs. “Standard Fe” group (25.5 ± 0.8 mg and 23.3 ± 0.6 mg, respectively, P < 0.05, Figure 1).

### Gene expression of iron transporters (DMT-1, Ferroportin) and DcytB in the duodenum

Gene expression analysis of duodenal DMT-1, Ferroportin and DcytB, with results reported relative to 18S rRNA, indicated no significant differences in mRNA expression of DMT1, DcytB and ferroportin between treatment groups (n = 6, P > 0.05).

### Caco-2 cell ferritin formation

An *in vitro* digestion/Caco-2 cell culture model was used to evaluate Fe bioavailability from the test diets by measuring ferritin formation in the cells (ie. a measure of cell Fe uptake) following exposure to digests of the samples. Ferritin concentrations, which are the measure of Fe uptake and bioavailability in this model, were not different in cells exposed to the “Biofortified Fe” vs. “Standard Fe” beans only and bean based diets (n = 6, Table 2).

**Table 2:** Ferritin concentrations in Caco-2 cells exposed to samples of beans only (whole bean) and bean-based diets

**Tested sample**^**1**^	**Ferritin (ng/mg of protein)**
“Standard Fe” bean only (59 μgFe/g bean)	2.31 ± 0.11^c^
“Biofortified Fe” bean only (88 μgFe/g bean)	2.19 ± 0.14^c^
“Standard Fe” bean-based diet (39 μgFe/g diet)	2.97 ± 0.10^b^
“Biofortified Fe” bean-based diet (53 μgFe/g diet)	2.75 ± 0. 09^b^
Baseline^2^	3.28 ± 0.139^a^

^1^Caco-2 bioassay procedures and preparation of the digested samples are described in the materials and methods sections.

^2^Cells were exposed to only MEM growth media without added food digests and Fe. Values are means ± SEM, n = 6.

^a,b,c^ Within a column, means without a common letter are different, P < 0.05.

### Ferritin and iron in the liver

The avian ferritins corresponded to a weight of approximately 470 to 500 kDa [7-9,24-26,28]. No significant differences in liver Fe or liver ferritin concentrations were measured between the treatment groups (n = 6, P > 0.05). The mean values of ferritin protein and the amount of iron present in the ferritin of the liver samples of all animals are presented in Table 3.

**Table 3:** **Ferritin protein and the iron**^**1**^**concentration in the liver**

**Treatment diet**	**Ferritin**	**Iron**	**Iron/Ferritin μmol**
	**μg/g wet weight**	**μg/g wet weight**	
“Biofortified Fe”	293 ± 11^a^	33.1 ± 2.2^a^	45.6 ± 3.5^a^
“Standard Fe”	282 ± 12^a^	27.2 ± 1.7^a^	39.8 ± 4.2^a^

^1^Atomic mass for iron is 55.8 g/mol.

^a^Values are mean ± SEM, n = 14. Within a column, means without a common letter are different, P < 0.05.

### Total phenolic concentration in the diet sample

Concentrations of the polyphenols known to be most prevalent in black beans were measured and are shown in Table 4. Overall it appears that kaempferol, epicatechin, myricetin, kaempferol 3-glucoside, and quercitin 3-glucoside were significantly higher in the high Fe beans.

**Table 4:** **Concentrations of the most prevalent polyphenols observed in the black beans**^**1**^**(nmol/g)**

**Compound**	**“Biofortified Fe”**	**“Standard Fe”**
Caffeic acid	6.0 ± 0.9^b^	25.8 ± 4.7^a^
Gallic acid	125.0 ± 8.8^a^	103.3 ± 17.8^a^
Ferulic acid	153.0 ± 11.1^a^	162.5 ± 20.1^a^
Kaempferol	5.0 ± 0.1^a^	0.00 ± 0.00^b^
Catechin	668.8 ± 31.1^a^	367.5 ± 25.5^b^
Myricetin	24.0 ± 1.7^a^	12.5 ± 4.0^b^
Kaempferol 3-glucoside	198.0 ± 10.7^a^	19.2 ± 5.6^b^
Quercetin 3-glucoside	239 ± 20.3^a^	45.8 ± 7.9^b^

^1^Analysis procedures of beans samples are described in the materials and methods sections.

^a,b^ Within a row, means without a common letter are different (n = 6, P < 0.05).

### Phytate:Fe molar ratios in the diet samples

The concentrations of phytic acid (IP_1–6_) and Fe in the diets were used to calculate the phytate to Fe molar raios. The ratios of phytate:Fe did not differ between diets and were 8.25 ± 0.65 and 8.95 ± 0.72 for the “Standard Fe” and “biofortified-Fe” bean based diets, respectively (n = 5; P > 0.05, Table 1).

## Discussion

In terms of biofortification, target levels for bean Fe concentration have been set at approximately 90 μg/g or higher, which should likely represent a 40 μg/g differential from more typical common bean Fe levels [29,30]. That target value is based on calculations that assume the percent bioavailability of the Fe is similar between the normal and enhanced lines, hence delivering more absorbable Fe. This assumption is potentially disastrous in terms of planning and ultimately moving forward with a human efficacy trial, as such trials can easily cost $500,000 or more. Given this expense, it is prudent to utilize inexpensive screening tools that are validated to predict relative differences in Fe bioavailability.

A strong case can now be made for utilizing the widely accepted and inexpensive *in vitro* digestion/Caco-2 cell culture model to determine if biofortified lines warrant advancement to human study [7-9]. Furthermore, this *in vitro* approach has recently been coupled with a poultry model for Fe bioavailability and it has confirmed the *in vitro* predictions [7-9]. Such observations certainly provide confidence in advancing lines for the more expensive human trials. If consistent correlations between these screening tools and human results can be established, then future work can be guided by these models thus enhancing the effectiveness of research dollars. Moreover, such lines will have to be monitored once released, and these models represent tools that will be needed to do so.

In the present study, the *in vitro* results predicted that the biofortified (high Fe) bean will not provide additional absorbable Fe. The *in vivo* results reflect this prediction, as no significant differences in liver ferritin and liver Fe were observed, and only slight differences were observed in Hb (higher only on day 42 in the high Fe bean group) and total body Hb (also higher on day 42 for the high Fe bean group). Hemoglobin maintenance efficiency was higher in the low Fe group at days 14th, 21st and 28th, indicating an adaptive response (ie. a relative up regulation of absorption) to less absorbable dietary Fe. The duodenal relative mRNA abundance analysis of DMT-1, DcytB and ferroportin that showed no significant differences between the standard (Low Fe) group vs. the biofortified (High Fe) group (P > 0.05), suggests a lower dietary Fe bioavailability in both groups [5-9]. Taken together, all of the above mentioned measures of Fe status indicate that the two dietary treatment groups were of relatively similar Fe status, with only a hint of more absorbable Fe being provided by the high Fe black beans.

The strong inhibition of Fe uptake reflected in the *in vitro* results is typical of a strong polyphenolic inhibition of Fe bioavailability. Similar results (ie. when the Caco-2 cell ferritin formation is at or below the untreated baseline values), have been seen many times in the use of this in vitro model [7,8,21]. The polyphenolic profile and measurement of concentration indicates that the high Fe beans were also higher in polyphenolic content, specifically, catechin, myricetin, kaempferol 3-glucoside and quercetin 3-glucoside (Table 4), than the low Fe beans. Thus it appears that the potential nutritional benefit from the higher Fe content was offset by the increased levels of polyphenolics. These observations clearly suggest that further research should be done to explore the metabolic and potential genetic link between Fe content and polyphenols.

A wealth of information is available on the phenolic and polyphenolic profile of foods [31-34], and specifically in regards to beans [27,35]. In addition, there are a considerable number of papers that focus on the Fe-binding properties of polyphenols as related to antioxidation [36]. Perron et al. [37] investigated the kinetics of Fe oxidation upon binding to polyphenols and observed that gallol-containing polyphenols have faster rates of Fe oxidation (ie. Fe^+2^ to Fe^+3^) than their catechol analogs. This faster rate of oxidation was believed to be related to the strength of the Fe binding. Thus, compounds such as epicatechin and quercitin would be expected to have slower rates of Fe oxidation and less strength of Fe binding than their respective gallol analogs, epigallocatechin and myricitin. These differences could be translated into different effects on Fe bioavailability as greater oxidation and stronger binding of Fe is generally correlated with lower bioavailability. However it is important to note that in the above studies, conditions for the measured reactions were not designed to match the conditions of the intestinal lumen, hence the oxidation and binding of Fe could differ.

In regards to Fe bioavailability there is clear evidence that polyphenolic compounds related to seed coat color in beans have a strong inhibitory effect on Fe bioavailability. Several in vitro and in vivo studies have demonstrated that Fe bioavailability from black, red and pinto beans is significantly less than that of white beans [5-8,21,38]. To date there has only been one published study that has linked specific bean seed coat polyphenols to inhibition of Fe bioavailability [38]. In that study the flavonoids kaempferol and quercitin identified in red, pinto and black bean hulls were shown to inhibit Fe bioavailability. This work did not exclude any other compounds from having an effect on Fe bioavailability, it merely documented inhibitory effects of these compounds which were found in high quantities in the red and pinto bean seed coats, to a lesser extent in black beans, and absent from white beans.

An interesting study by Lin et al. [27] profiled but did not quantify the polyphenolics in 24 bean samples representing 17 varieties and 7 “generic off the shelf” samples, with the total group belonging to 10 commercial US market classes. Their work showed that the beans could be organized into 6 classes based on their polyphenolic profiles. The classes were black, pinto, pink, light red kidney bean, small red and navy. In general, these groups contained similar hydroxycinnaminic acid derivatives as their main phenolic profile with the flavonoid content less prominent but more varied and distinctive for each group. For each group there were 10 or less flavonoid compounds that were predominant. If one were to screen these for effects on Fe bioavailability then certainly a relatively high throughput system would be needed, as concentration and interactive effects would also need to be considered. In our opinion, the *in vitro* digestion/Caco-2 model [23] or a variation thereof is capable of performing this work.

Recently, we were able to demonstrate that specific polyphenols found in black bean seed coats are inhibitors of Fe uptake whereas others are promoters of Fe uptake [39]. This work uses a modification of the *in vitro* digestion/Caco-2 cell model [23] and LC/MS to measure relative amounts of flavonoids and hydroxycinnamic acids in black bean seed coats that were previously identified as major polyphenolic components of common bean [27]. The aforementioned research has enabled us to generate a key list of phenolic and polyphenol compounds in black beans that predominate and may be related to Fe bioavailability. Those compounds are reported in Table 4. Comparisons of the polyphenols between the two lines used in this study certainly indicate that the higher polyphenols in the high Fe line may have negated the nutritional benefit of higher Fe. These differences could be environmental in origin, or possibly linked to the effort to breed for higher Fe. Regardless, these results indicate that the polyphenolic profile of beans should be considered for future human efficacy studies of high Fe beans.

The list of polyphenols and flavonoids reported here represent the predominant compounds that were detected in the tested black beans, however, these compounds are part of a major group of compounds observed in black beans as previously reported [27]. As it was shown before, several of the detected compounds present Fe^+3^ binding ability that results in decreased Fe bioavailability and absorption [34-37,40]. For example, quercetin, which is one of the most common flavonols present in nature and is the predominant flavonoid in the diet, and was detected in increased concentrations in the biofortified Fe bean variety. Quercetin was shown to be a strong Fe^+3^ chelator, hence, suggested to decrease Fe bioavailability in foods [40]. In addition the compound catechin, that was also detected in increased concentration in the biofortified Fe bean variety was also reported to have strong binding affinity to Fe^+3^, specifically in a less acidic environment, as in the duodenum where most absorption of Fe occurs [41]. Increased concentrations of kaempferol 3-glucoside were also detected in the biofortified Fe black bean variety. Previously, it was demonstrated that kaempferol 3- glucoside in red and pinto bean seed inhibited Fe bioavailability *in vitro*[38]. Thus, seed coat kaempferol was identified as a potent inhibitory factor affecting iron bioavailability in the red and pinto beans studied. Results comparing the inhibitory effects of kaempferol, quercitrin, and astragalin on iron bioavailability suggest that the 3′,4′-dihydroxy group on the B-ring in flavonoids contributes to the lower iron bioavailability [38].

In summary, the current study suggests that increasing Fe concentrations in black beans by about 30 μg/g provides a very small increase in the amount of bioavailable and therefore absorbable Fe *in vivo*. The increased polyphenol concentration in the high Fe beans indicates that these compounds must also be considered when advancing high Fe lines for human studies in order to achieve significant nutritional benefits.

## Conclusion

Based on the data shown here, the *in vivo* results suggested a relatively small nutritional benefit to the biofortified bean variety. Evidence suggests that the nutritional benefit of the biofortified beans is reduced by the presence of polyphenols. Hence, modification of the bean seed coat polyphenols may be a means to improve bean Fe bioavailability. We conclude that biofortified black beans remain are a promising vehicle for increasing intakes of bioavailable Fe in populations that consume these beans as a dietary staple. However, the bean polyphenols profile must be further evaluated in order to improve the nutritional benefit of beans.

## Endnotes

Mention of a trademark, proprietary product or vendor does not constitute a guarantee or warranty of the product by the United states Department of Agriculture and does not imply its approval to the exclusion of other products or vendors that may also be suitable.

## Abbreviations

Fe: Iron; Hb: Hemoglobin; Hb- Fe: Hemoglobin iron; HME: Hemoglobin maintenance efficiency; BW: Body weight; CIAT: Centro Internacional de Agricultural Tropical; PCR: Polymerase chain reaction; DMT-1: Divalent metal transporter 1; DcytB: Duodenal cytochrome B; Da: Dalton; MEM: Minimum essential media.

## Competing interests

The authors declare that they have no competing interests.

## Authors’ contributions

ET was a co-principal investigator in developing the study protocol and design, performing the data collection, statistical analyses, *in vivo* and *in vitro* analyses, and writing of the manuscript. SB provided the bean lines used in the study. SR assisted with the *in vivo* data collection and analysis. JH performed the polyphenolic bean analysis. RG was a co-principal investigator who participated in the study design, in vivo and in vitro analyses, and edited the manuscript. All authors approved the final manuscript.
